# Occlusion abdominale révélant un lymphome colique: à propos d’un cas

**DOI:** 10.11604/pamj.2024.47.219.42319

**Published:** 2024-04-29

**Authors:** Mohamed Zioui, Fatim Ezzahra Lahlimi, Illias Tazi

**Affiliations:** 1 Service d’Hématologie et Greffe de Moelle, Centre Hospitalier Universitaire Mohammed VI, Faculté de Médecine et de Pharmacie, Université Cadi Ayyad, Marrakech, Maroc

**Keywords:** Caecum, lymphome, occlusion, urgence, cas clinique, Cecum, lymphoma, occlusion, emergency, case report

## Abstract

Le lymphome du colon est une tumeur gastro-intestinale maligne rare qui peut être révélé par une complication exceptionnelle et grave qui est l´occlusion intestinale. Son traitement repose sur la chirurgie et l´immunochimiothérapie. Nous rapportons le cas d´un lymphome diffus à grandes cellules B colique révélé par une occlusion, diagnostiqué sur une pièce opératoire chez un homme de 64 ans qui a été en rémission complète après six cures R-CHOP.

## Abstract (en)

Colonic lymphoma is a rare malignant gastrointestinal tumor that can be revealed by an exceptional and serious complication: intestinal obstruction. Treatment is based on surgery and chemotherapy. We here report a case of diffuse colonic large B-cell lymphoma revealed by occlusion and diagnosed based on the examination of surgical specimen in a 64-year-old man who was in complete remission after six courses of R-CHOP.

## Introduction

Le colon est une localisation rare du lymphome [1], représentant seulement 0,3% des néoplasies du colon et environ 3% des lymphomes gastro-intestinaux [2]. Le caecum comporte un tissu lymphatique très important, ce qui en fait la localisation la plus fréquente du lymphome gastro-intestinal [3]. Sur le plan anatomopathologique, le lymphome diffus à grandes cellules B (LDGCB) reste le sous-type le plus fréquent [4]. L´atypie de son tableau clinique ainsi que sa localisation inhabituelle rend le diagnostic difficile et tardif d´où installation de complications graves, telle que l´occlusion colique, qui peut engager le pronostic vital [5]. Nous rapportons le cas d'un LDGCB colique révélé par une occlusion chez un homme de 64 ans, et nous voulons à travers ce cas mettre la lumière sur cette présentation rare et grave du LDGCB.

## Patient et observation

**Informations relatives aux patients:** un patient âgé de 64 ans, sans antécédents pathologiques particuliers, qui s´est présenté pour des douleurs abdominales généralisées d´installation progressive associées à des vomissements intermittents et des rectorragies de faible abondance dans un contexte d'apyrexie et d'altération de l´état général.

**Résultats cliniques:** les constantes vitales étaient normales. L'examen de l'abdomen ainsi que le toucher rectal étaient normaux.

**Figure 1:**
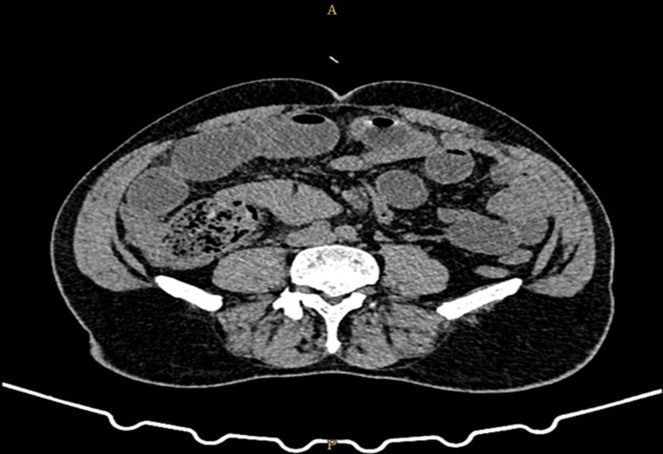
scanner abdominopelvien montrant un épaississement du caecum

**Démarche diagnostique:** une coloscopie réalisée a montré un processus bourgeonnant et dur de la valvule de Bauhin. L'examen anatomopathologique de ce processus a montré la présence d´une lésion inflammatoire chronique en poussée aiguë ulcérée, renfermant de rares amas cellulaires denses dont la rareté n'a pas permis de préciser avec certitude leur nature. Un scanner abdominopelvien a été réalisé et a objectivé un important épaississement circonférentiel bourgeonnant du caecum étendu de la jonction iléo-caecale de 21 mm d'épaisseur maximal, étendu sur 8 cm de hauteur (Figure 1). Cet épaississement s´accompagne d´une infiltration de la graisse de voisinage avec la présence de nodules péritonéaux de voisinage et d'un magma d'adénopathies mésentérique atteignant 24 mm de petit axe pour la plus grosse.

**Chronologie:** quatre jours après, le patient a présenté un syndrome occlusif fait d´arrêt de matières et de gaz avec vomissements alimentaires incoercibles dans un contexte d´apyrexie et d´altération de l´état général. La radiographie sans préparation de l'abdomen a confirmé le diagnostic de l´occlusion colique. Le patient a été opéré et l´exploration a découvert un épanchement séreux de faible abondance avec la présence d´une tumeur du carrefour iléo-caecale de 5x4 cm, mobile et sténosante avec également, la présence d´adénopathies mésocoliques droites. Le patient a bénéficié d´une hémicolectomie droite avec une anastomose iléocolique mécanique.

**Figure 2:**
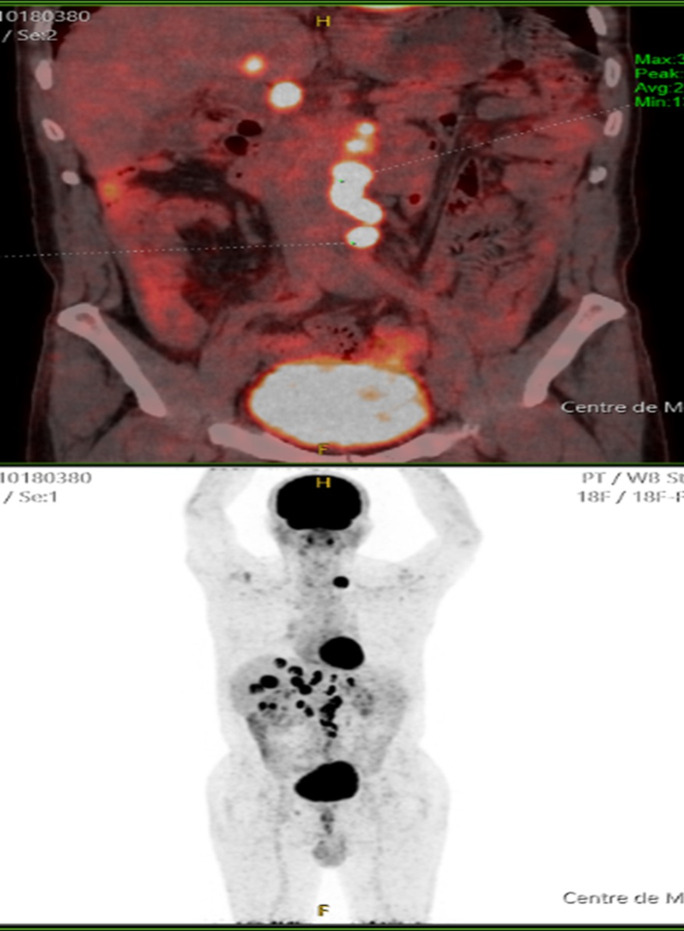
TEP-SCAN mettant en évidence l´extension lymphomateuse au niveau ganglionnaire sus et sous diaphragmatique, au niveau hépatique, péritonéal et pancréatique

L´anatomopathologie de la pièce de l´hémicolectomie a révélé la présence d'une prolifération tumorale malignes à cellules rondes panpariétale de densité cellulaire modérée à élevée. Le curage ganglionnaire a ramené 16 ganglions lymphoïdes dont sept sont tumoraux. Un complément immunohistochimique a confirmé le diagnostic d´un lymphome malin non hodgkinien B diffus à grande cellules de type centre germinatif avec anticorps anti CD20 positif, anti BCL2 positif, anti CD 10 positif, anti CD 3 positif, anti pan-cytokératine négatif et anti-chromogranine négatif. L'anticorps anti- Ki 67 était positif sur 70% des cellules marquées. Un TEP-scan a montré une extension lymphomateuse au ganglionnaire sus et sous diaphragmatique, hépatique, péritonéal et pancréatique (Figure 2). Une fibroscopie oesogastroduodénale (FOGD) a été réalisée et n´a montré aucune anomalie.

**Intervention thérapeutique:** la décision thérapeutique était de mettre le patient sous six cures de protocole R-CHOP (Rituximab, Cyclophosphamide, Doxorubicine, Vincristine et Prednisone).

**Suivi et résultats des interventions thérapeutiques:** le patient a présenté une bonne évolution clinique et radiologique avec le TEP-scan réalisé en fin de traitement montrant une rémission métabolique majeure.

**Perspectives du patient:** le patient est satisfait de sa prise en charge globale.

**Consentement éclairé:** il a été obtenu du patient pour que nous puissions utiliser pour ce rapport de cas.

## Discussion

L'occlusion peut révéler 8 à 29% des néoplasies coliques et plus rarement des lymphomes [1,6]. Donc, le diagnostic étiologique de l´occlusion colique nécessite la prise en considération de l'éventualité de la découverte d´un lymphome à côté des autres étiologies les plus fréquentes. La majorité des lymphomes coliques sont des lymphomes non hodgkiniens (LNH), et le lymphome diffus à grandes cellules B est le sous-type le plus fréquent [4]. Bien que l'étiologie reste inconnue, certains facteurs de risque décrits peuvent être considérés comme les antécédents de lymphome, les traitements antérieurs par radiothérapie ou chimiothérapie, l'immunodépression, les maladies auto-immunes ou certaines infections virales [7]. Les lymphomes coliques peuvent se manifester par des douleurs abdominales, constipation, diarrhée, méléna, symptômes B (fièvre, sueurs nocturnes, perte de poids) et, dans de rares cas, de perforation colique [8]. La localisation la plus fréquente du lymphome colique est iléo-cæcale [9], ce qui était le cas de notre patient.

L'examen d'imagerie le plus courant pour l'étude des lymphomes coliques est le scanner. Il permet de différencier, selon les caractéristiques scannographiques entre les lymphomes et les adénocarcinomes du colon [10]. Toutefois, le diagnostic du lymphome colique est fréquemment différé dans la plupart des cas. Ce retard est dû à la symptomatologie peu spécifique du lymphome colique et le diagnostic est généralement basé sur des découvertes fortuites. Le TEP-scan est indiqué pour stadifier la maladie et pour exclure une dissémination du lymphome [11]. La coloscopie avec biopsie reste le moyen d'exploration diagnostique de choix dans les lymphomes colorectaux. Une atteinte pariétale infiltrante circonférentielle et sténosante majeure peut être responsable d'occlusion aiguë [12]. Chez notre patient, malgré que la lésion fût à hauteur de 8 cm de diamètre, sa biopsie n´était pas concluante.

L'étude histologique est indispensable au diagnostic. Elle a été réalisée sur une pièce opératoire chez notre patient. Morphologiquement, le LDGCB est constitué de cellules lymphoïdes atypiques de grande taille avec des nucléoles proéminents et un cytoplasme basophile qui ont un schéma de croissance diffus oblitérant l'architecture des glandes coliques. L'immunohistochimie et la cytométrie en flux confirment l'immunophénotype du LDGCB. Les cellules tumorales expriment généralement des marqueurs de cellules pan B tels que CD20, CD19, CD 22, CD45 et CD79a [13]. Le pronostic et la prise en charge des lymphomes gastro-intestinaux dépendent de la stadification. Les patients aux stades I et II ont une survie globale plus longue que ces aux stades III ou IV [11,13,14]. Dans notre cas, les examens ont conclu à un stade VI disséminée selon Ann Arbor modifiée par Musshoff pour le tube digestif.

Malgré l'absence de consensus sur le traitement du LDGCB gastro-intestinal, l'option thérapeutique la plus optimale est la résection chirurgicale suivie d'une immunochimiothérapie à base de Rituximab plus CHOP (R-CHOP) [14]. La chirurgie reste indiquée devant le risque élevé de complications (une occlusion, une perforation ou une hémorragie). En l'associant à la chimiothérapie, elle a permis d´avoir des résultats satisfaisants avec un taux de rechute plus faible qu´avec une chimiothérapie seule [15]. Chez notre patient, l'occlusion colique d'avant le diagnostic définitif a nécessité une intervention chirurgicale d'urgence par une laparotomie de sauvetage permettant de confirmer le diagnostic de LDGCB. Un traitement d'immunochimiothérapie basé sur le protocole R-CHOP a été mis en place en aval avec une suite favorable et une bonne évolution clinique et paraclinique.

## Conclusion

L'occlusion colique est une urgence diagnostique et thérapeutique qui nécessite une prise en considération d´une éventuelle origine tumorale dont le lymphome. Toutefois, ce diagnostic est souvent différé causant le retard de prise en charge vu sa symptomatologie peu spécifique. Le traitement chirurgical avec la chimiothérapie postopératoire sont les piliers de la prise en charge.

## Remerciements

Nous remercions Dr Ouadii Abakarim d´avoir contribué à la réalisation de ce travail ainsi que le staff médical.
